# Nasal Injuries Related to Respiratory Support Interfaces in Preterm Infants: Neonatal Course and 12-Month Outcome

**DOI:** 10.3390/children12070840

**Published:** 2025-06-26

**Authors:** Marielle Jamaux, Corisande Gibier, Laurence Dillenseger, Gwenaelle Fourie, Claire Langlet-Muteau, Jennifer Rondel, Jacqueline Matis, Bénédicte Matz, Valérie Schmitt, Nicolas Meyer, Pierre Kuhn, Claire Zores

**Affiliations:** 1 Médecine et Réanimation du Nouveau-né, Service de Pédiatrie 2, Pôle Médico-Chirurgical Pédiatrique Hôpital de Hautepierre, Centre Hospitalier Universitaire de Strasbourg, Avenue Molière, 67098 Strasbourg, France; marielle.jamaux@chru-strasbourg.fr (M.J.); jacqueline.matis@chru-strasbourg.fr (J.M.); pierre.kuhn@chru-strasbourg.fr (P.K.); 2 LNCA, UMR 7364, CNRS and University of Strasbourg, 67081 Strasbourg, France; 3 Neonatal Research Unit, Department of Women’s and Children’s Health, Karolinska Institute, 171 77 Stockholm, Sweden

**Keywords:** continuous positive airway pressure, nasal injury, non-invasive ventilation, preterm infants, respiratory support, skin injury

## Abstract

*Background*: Respiratory support required by preterm infants involves contact between their immature skin and ventilation devices, which can lead to skin breakdown. *Methods*: A prospective observational study including newborns with a nasal injury related to respiratory support, born at gestational age < 33 weeks, from May 2020 to January 2022, in the neonatal intensive care unit of Strasbourg. Injuries were recorded using a validated scale at inclusion and 3, 7 and 28 days. Sequelae were evaluated at discharge and 4, 9 and 12 months post-menstrual age. *Results*: In total, 64/276 newborns (23%) had a nasal injury. Most of the injuries were stage 2 (34/64, 53%) and stage 1 (25/64, 39%). The interface most frequently associated with injury was continuous positive airway pressure (53/64, 83%). Favorable evolution was associated with the injury site (*p* < 0.01) and the type of respiratory support needed when collecting at the 28th day (*p* = 0.04). At discharge, 34/58 infants (59%) had sequelae. The presence of a scar was associated with the maximum injury severity (*p* < 0.001) and total duration of respiratory support (*p* = 0.02). At 12 months, 31/47 infants (66%) had esthetic sequelae. *Conclusions*: Nasal injuries related to respiratory support in preterm infants were frequent, and more than half of the injuries resulted in medium-term sequelae.

## 1. Introduction

Very preterm birth is associated with an increased risk of respiratory distress or apnea of prematurity, which requires the provision of respiratory support to the infant. Continuous positive airway pressure (CPAP) is now the gold standard for non-invasive ventilation [1]. It reduces the need for invasive ventilation, which is a risk factor for bronchopulmonary dysplasia [2]. All respiratory support requires contact with the newborn’s skin, which is itself immature. The pressure exerted by various devices (intubation tubes, nasal masks, nasal prongs or nasal cannulas) on cutaneous and mucosal tissue can cause injuries of varying severity and location.

Injuries related to CPAP are the most extensively studied. Their incidence ranges from 20% to 100%, depending on the population, material and care strategy [3,4,5,6,7,8]. The severity of injuries, defined by the Fischer et al. classification [9], remains relatively identical across studies. Stage 1 is the most common, representing 80% of injuries, and is described as persistent erythema on otherwise intact skin. Stage 2 corresponds to superficial ulceration or erosion, with partial-thickness skin loss. Stage 3, characterized by necrosis with full-thickness skin loss, represents less than 1% to 11% of injuries. Injuries typically appear 2 to 3 days after CPAP initiation for cutaneous injuries [4,9] and 8 to 9 days for mucosal injuries [10]. Known risk factors for the occurrence of an injury are birth before 30 weeks’ gestational age, birth weight < 1500 g, prolonged respiratory support, the opacity of the device, the degree of skin immaturity, incubator humidity, the insufflation air heater temperature and the frequency of changes in the infant’s position [3,6,11,12,13]. Hydrocolloid skin protection applied to the skin in contact with the respiratory support device has been found to be an effective preventive measure [14,15]. Nasal masks cause fewer nasal injuries than nasal prongs [6,16,17]. Finally, regular and rigorous monitoring of the skin condition helps in detecting nasal injuries earlier and at an earlier stage [18].

To the best of our knowledge, skin injuries related to other ventilation strategies, such as intubation tubes and the Ram cannula, have been reported in only isolated cases. The frequency of nasal injuries related to high-flow nasal cannulas has only been compared with those related to CPAP [19].

Moreover, although the incidence of nasal injuries due to CPAP has been well studied, their evolution has been poorly documented. Only a few studies have described the possible sequelae of injuries, identifying esthetic and functional complications [20,21]: hyperemia, structural changes to the nose, nasal obstruction and necrosis, particularly of the columella or septum, which may require surgical treatment.

We conducted this prospective observational study to describe nasal injuries related to contact with all respiratory support interfaces and the incidence of such injuries in preterm infants, as well as their evolution. Secondary outcomes were the short- and medium-term evolution of these injuries and factors associated with favorable or unfavorable evolution.

## 2. Materials and Methods

### 2.1. Design

This prospective and observational study was conducted from 1 May 2020 to 1 January 2022, in the neonatal intensive care unit of University Hospital of Strasbourg.

### 2.2. Participants

All preterm infants born before 33 weeks of gestational age, who were hospitalized at the University Hospital of Strasbourg and received respiratory support, were eligible. We included newborns with a nasal injury related to contact with a respiratory support. In the case of multiple injuries, if these were independent and occurred at different times, the infant could be included a second time. If the injuries occurred simultaneously or were associated, the injury occurring first or with the maximum severity was defined as the main injury.

### 2.3. Routine Care and Material

At the time of the study, as part of routine care, nurses monitored the skin’s condition every time they cared for the newborn (i.e., every 3 or 4 h). The choice of respiratory support for each newborn depended on clinical stability and was at the discretion of the medical team. The types of intubation tubes were silicone tubes (Portex, Smiths Medical, Minneapolis, MN, USA; Teleflex Medical, Kamunting, Malaysia), nasal masks (Infant Flow, Vyaire Medical, Mettawa, IL, USA) and nasal prongs (Infant Flow, CareFusion, Yorba Linda, CA, USA). The choice to use a mask or prongs was at the discretion of the nurse in charge of the infant; in practice, whenever possible, the type of interface was alternated, as was its size. The cannulas used were the Ram cannula (Neotech Products, Valencia, CA, USA) and high-flow nasal cannulas (Optiflow Fisher and Paykel Healthcare, Auckland, New Zealand). The choice of interface size was based on the use of an abacus supplied by the manufacturer. In the event of a nasal injury, a secondary protective measure (hydrocolloid, change in interface, change in ventilatory mode) was applied on a case-by-case basis. No hydrocolloid prophylaxis was used from birth.

### 2.4. Protocol Description

A nasal injury assessment and monitoring tool was developed for the study (Supplementary Materials: Document S1). It included a scheme to indicate the precise site of the injury and a table to record the injury severity, following the Fischer et al. classification [9]. The presence or absence of skin loss, dilatation or enlargement of the nostril and the use of a protective measure were noted. This tool was introduced to the medical and nursing teams during dedicated sessions.

In the event that a nasal lesion appeared and was reported, the newborn was included, and the data collection form was completed by the nurse or physician in charge of the newborn, in collaboration with the parents. Parental consent was obtained before study enrolment. Data were collected on the following: date of occurrence of the injury; duration, type, and ventilation interface used at the time of the injury; and gestational age and actual age of the infant. The injury was then monitored at 3, 7 and 28 days after inclusion and at discharge by the nurse or physician in charge of the newborn. A picture of the injury was taken at each step to confirm the severity a posteriori. Progression was defined as favorable with a change to a lower severity stage or stabilization for a stage 1 injury, and it was defined as unfavorable with progression to a higher severity stage, stabilization for stage 2 and 3 injuries, the occurrence of dilatation or nostril asymmetry or the involvement of several sites.

If the newborn was transferred to another hospital, follow-up was maintained, and the data collection form was completed by the medical and nursing teams at these hospitals.

The medium-term evolution was studied at 4, 9 and 12 months post-menstrual age during specific follow-up consultations for preterm infants. The following data were collected: site of any scarring; change in nasal appearance; loss of substance, dilatation or enlargement of the nostril; change in skin pigmentation or thickness; respiratory noises; or deviation of the nasal septum. At the first follow-up visit, a questionnaire was submitted to parents to collect their perceptions of possible sequelae in the child (Supplementary Materials: Document S2). If consultations were not attended, parents were contacted by phone or e-mail.

### 2.5. Factors Associated with Injury Evolution

Demographic data were extracted from the infant’s medical chart: gestational age, sex, Apgar score at 1 and 5 min, birth weight and possible classification as small for gestational age (weight < 10th percentile according to AUDIPOG) [22], maternal antenatal corticosteroids used, mode of delivery, skin phenotype (according to the Fitzpatrick classification [23]), total duration of invasive ventilation, ventilation by CPAP, Ram cannula and high-flow nasal cannula, total duration of mask and prong use for CPAP, incubator humidification rate at inclusion, temperature of insufflated air heating and humidification system at inclusion and duration of treatment of central apnea with caffeine and doxapram. We also recorded the occurrence of complications such as bronchopulmonary dysplasia, intraventricular hemorrhage, periventricular leukomalacia and retinopathy of prematurity.

When a nasal injury was identified, any implemented secondary protective measures were recorded: change in interface used, application of a hydrocolloid, switch to another respiratory support or other measures (local care with breast milk and application of emollient or dressings).

### 2.6. Statistical Analysis

The Statistica software 12 was used. Sample normality was determined by the Shapiro–Wilk test. Data with a Gaussian distribution are presented as means (SD) and those with a non-Gaussian distribution as the median (range) or number (percentage). For categorical data, univariate statistical analyses involved chi-squared or Fisher’s exact tests, and, for continuous data, the Student *t*, Kruskal–Wallis or Mann–Whitney test was used depending on the normality of the sample. A two-sided *p* < 0.05 was considered statistically significant.

### 2.7. Ethical Approval

This study was approved by the ethics committee of the faculties of medicine, odontology, pharmacy, nursing, physiotherapy and maieutics of the University Hospital of Strasbourg (3 March 2020; reference FC/2019-87). Parents were informed about the study when a nasal injury was discovered in their infant. Both parents provided written informed consent for their infants to participate in the prospective recording of medical data in the hospital’s database at the neonatal intensive care unit, which was registered at the National Commission on Informatics and Liberty (CNIL) of France.

## 3. Results

### 3.1. Characteristics of Studied Infants

**Figure 1:**
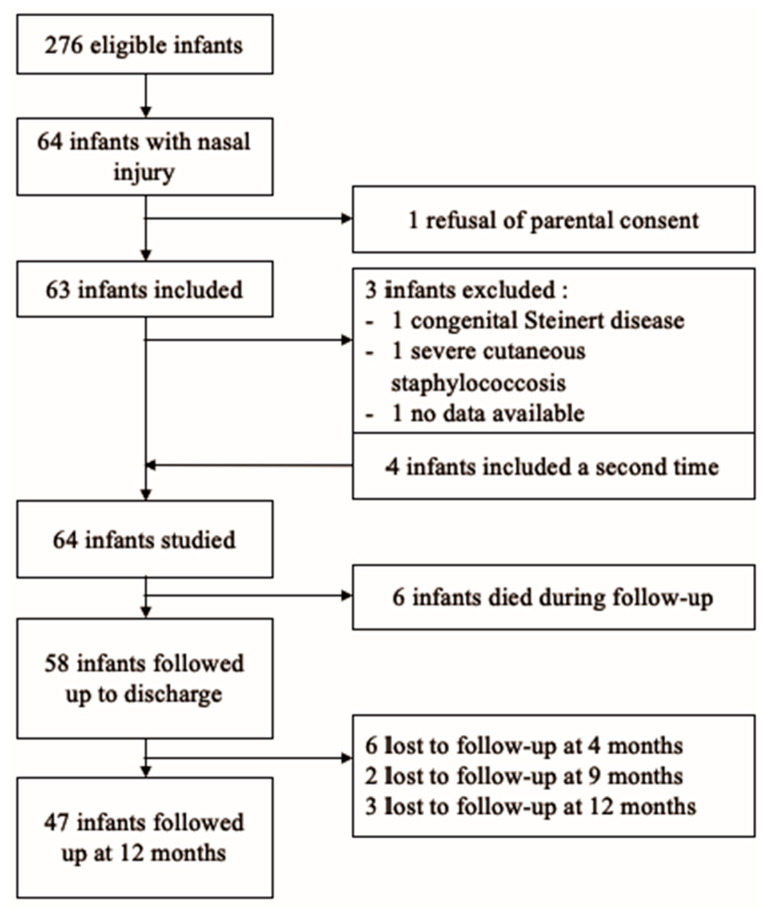
Flow chart.

The incidence of nasal injuries related to contact with a respiratory support interface was 23.2% (64/276) (see Figure 1). The patient characteristics (n = 64) referring to children with skin damage are summarized in Table 1. The median gestational age of the infants was 27^6/7^ weeks (range 23^4/7^ to 32^6/7^), and the median birth weight was 986 g (485 to 2090 g). At 1 year post-menstrual age, 47 infants were followed up with, and the lost-to-follow-up rate from discharge was 19%.

### 3.2. Nasal Injuries at Inclusion

In total, 25/64 injuries were stage 1 (39.1%), 34 were stage 2 (53.1%) and five were stage 3 (7.8%) (Supplementary Materials, Table S1) and appeared at a median [minimal; maximal] of 11 [1;49] days of life. Moreover, 53 injuries were localized to a single site, and 11 (17.2%) were associated with secondary sites of injuries with an equal or lower stage. When the injuries affected two to three sites simultaneously, the most severe lesion was recorded. All parts of the nose were examined, and all persisting lesions were reported and would have been mentioned in case of persistence, but this was not the case for secondary lesions. The site of injury differed significantly by the ventilatory support used (*p* = 0.001). Injuries associated with invasive ventilation—typically occurring in our center by the nasal route—were mainly located in the internal part of the nostril orifice (81.8% of injuries), whereas those occurring with CPAP involved various sites—primarily the columella (37.3% of injuries), followed by the intranasal mucosa (20.9%) and the internal part of the nostril orifice (17.9%). An extranasal site was described in the forehead of an infant receiving CPAP. The injury, which occurred with high-flow nasal cannulas, was stage 1 and involved the internal part of the nostril orifice. Of the 64 injuries, 53 occurred with CPAP (82.8%) and 10 (15.6%) with invasive ventilation (as shown in Supplementary Materials, Table S1) and one with a high-flow nasal cannula. No ventilatory support, such as a Ram cannula, was used during the study period. Among newborns with CPAP ventilation, 36/53 had a mask interface when the injury was diagnosed (67.9%) and 17/53 (32.1%) had nasal prongs (Supplementary Materials, Table S2). All neonates with injuries associated with nasal prongs benefited from alternating the interface between the prongs and masks. Injury severity was not significantly associated with the ventilatory mode responsible for the injury (*p* = 1).

Tube-related injuries were detected after a median duration of respiratory support of 9 days (range 1 to 38) and CPAP-related injuries after a median duration of respiratory support of 11 days (1 to 49); the one high-flow nasal cannula-related injury occurred after 7 days of respiratory support.

### 3.3. Nasal Injuries in the First Month of Follow-Up

Severity of Injuries

During the first month, 29/64 injuries (45.3%) worsened to stage 3 and 23/64 (35.9%) to stage 2.

Sites of Injury

**Figure 2:**
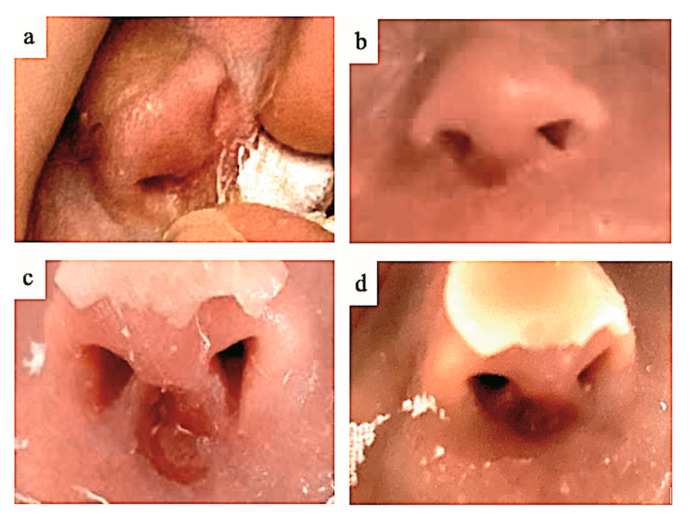
Evolution of a columella injury at inclusion (**a**), day 3 (**b**), day 7 (**c**) and day 28 (**d**).

Injuries of the columella remained the most frequent and represented 32% to 43% of all nasal injuries (see Figure 2, depicting the evolution of a columella injury in an infant at inclusion (a), day 3 (b), day 7 (c) and day 28 (d), for a representative child as an example). These were followed by injuries of the internal part of the nostril orifice (23–29%), the intranasal mucosa (18–21%) and the cartilaginous dorsum (7–13%).

Evolution of Injuries

In total, 22/64 injuries (34%) had evolved favorably by the third day, 29/63 (46%) at 1 week and 24/60 (40%) at 28 days (Table 2). Evolution at day 28 was significantly associated with the site of the initial injury (*p* = 0.009) and the type of respiratory support (*p* = 0.04).

Secondary protective measures were not significantly associated with injury evolution (Table 2). Injuries with and without secondary protective measures did not differ in severity (severity at inclusion and maximum severity). However, they did differ in the site protected. Indeed, the columella was protected more frequently than other sites (*p* = 0.04).

### 3.4. Nasal Injuries at Discharge

We followed 58 infants until discharge. The median age was 79 days (range 33 to 98), for a median post-menstrual age of 38^2/7^ weeks (36^0/7^ to 42^5/7^). In total, 34 infants (58.6%) had at least one sequela, and 17 (29.3%) had nostril dilatation or asymmetry. For more characteristics, see Table 3.

The presence of sequelae at discharge was significantly associated with the duration of ventilation (*p* = 0.02), particularly non-invasive (*p* = 0.005), and the maximum severity of injury (*p* < 0.001) (Table 3).

### 3.5. Medium-Term Evolution of Injuries

Evolution of Injuries

Medium-term follow-up involved 52 infants at 4 months post-menstrual age, 50 at 9 months post-menstrual age and 47 at 1 year post-menstrual age.

In total, 32/52 children (61.5%) had a scar at 4 months corrected age—12 of them at multiple sites. In two infants discharged from hospital with an injury, the scar had healed by 4 months (one on the internal part of the nostril orifice, one on the external part of the nostril orifice). Conversely, five infants for whom no sequelae were reported at discharge had scars at 4 months. No scars in infants had healed between the 4-month and 9- and 12-month follow-ups.

At 12 months post-menstrual age, 31/47 infants (66%) had a scar. Six infants had several affected sites, with a total of 37 injuries: 20 injuries (54%) were located on the columella, eight (21.6%) on the intranasal mucosa, three (8.1%) on the internal part of the nostril orifice, two (5.4%) on the external part of the nostril orifice and two (5.4%) on the cartilaginous dorsum; two (5.4%) were extranasal.

Esthetic and Functional Consequences

**Figure 3:**
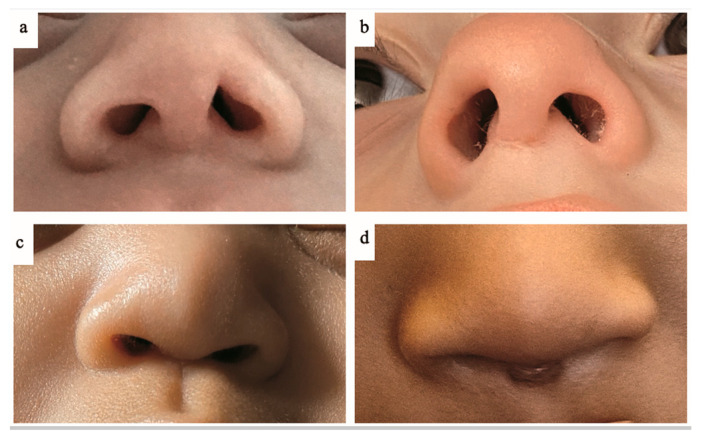
Sequelae of the internal part of the left nostril orifice (**a**), columella (**b**), internal part of the right nostril orifice and columella with deviation of the nasal septum (**c**), columella (**d**).

Among infants at 12 months corrected age, the esthetic sequelae identified were 25 (53.2%) losses of skin substance, excavation in the subcutaneous structures despite healing of the epidermis, 13 (27.7%) nostril dilatations or enlargements, 20 (42.6%) changes in skin pigmentation, 18 (38.3%) changes in skin thickness and two (4.3%) deviations of the nasal septum. None were associated with respiratory symptoms (see Figure 3 for examples of nasal injuries persisting at 1 year).

Parental Perceptions

A total of 46 questionnaires were completed by parents at 4 months post-menstrual age during the follow-up consultation; 12 parents (26.1%) had noticed a scar in their child, and seven (15.2%) had received comments from their family and friends about the scar. The main complaints were loss of substance for 10 parents (21.7%), dilatation or enlargement of the nostril for 12 (26.1%), changes in skin pigmentation for five (10.9%) and changes in skin thickness for eight (17.4%).

Parents of five children (10.9%) wished to have subsequent plastic surgery: three children had an isolated columella scar, and two had a columella scar associated with an intranasal injury. For three of the children, this desire for surgery was influenced by a comment made by family and friends.

## 4. Discussion

### 4.1. Main Results

In this study, nearly one quarter of newborns under 33 weeks of gestational age presented a nasal injury related to contact with a respiratory support interface, with most injuries occurring with CPAP. The main sites were the columella and the internal part of the nostril orifice. In half of the injuries, the severity was moderate from onset. Injuries progressively worsened over the first month. More than half of all injuries lead to sequelae at discharge, conditioned by the maximum severity of the injury and the duration of respiratory support. Esthetic sequelae at one year still affected more than half of the children. However, there were no functional sequelae.

### 4.2. Strengths and Limitations

The monocentric design of the study is one of its main limitations. In fact, the use of only one CPAP system during the study also limits the generalizability of the results and their comparison with other published papers in which other devices were used. Practices varying among centers could affect both the incidence and evolution of injuries. Moreover, because the type of interface is left to the clinician’s choice, management could differ among infants. Nevertheless, the completeness of the data collection; the prospective nature of this study with a large number of patients; the effective, medium-term and original follow-up; and the original features, such as the incidence of injuries by site and type of interface, all contribute to the strength of this study. Photographs taken at various stages of follow-up helped us to revalidate the injury severity stages a posteriori, thus limiting the risk of ranking bias.

### 4.3. Incidence of Nasal Injuries

The incidence of nasal injuries was comparable to that previously reported [6]. However, our study protocol included all types of respiratory support, unlike other studies, which only included CPAP. Our results are close to those of a Swiss study of 989 neonates with CPAP ventilation [9], in which the incidence of nasal injuries was 42.5% (77% in preterm infants under 32 weeks). The other cohorts were analyzed in geographically more distant countries, thereby suggesting an impact of the skin phenotype on injuries [24]. Nevertheless, the skin phenotype analysis in our study, including infants with a variety of ethnic origins, showed no difference in injuries.

### 4.4. Time of Occurrence and Severity

In contrast to the injury incidence, the time to injury occurrence and the severity of injuries in our study differed from those in other studies. In the study by Fischer et al., 88% of injuries were stage 1 and occurred within a mean of 2.7 days [9]. The association between a high degree of injury severity on diagnosis and a later time of injury occurrence may be explained by a failure to detect injuries at stage 1. Detecting erythema can be difficult, especially in the absence of a systematic nasal skin monitoring protocol. The absence of hydrocolloid application for primary prevention in our center may have been responsible for the greater injury severity, as several studies have demonstrated [25,26].

### 4.5. Factors Associated with Nasal Injury Occurrence

The care strategy—in particular, the type of interface used—and preventive measures seemed to play an important role in the development of injuries. In our center, with CPAP respiratory support, the nasal mask was most often used and was often alternated with nasal prongs when used. This strategy explains the higher number of injuries with a mask rather than prong interface in our study, although the former’s superiority in terms of safety and efficacy has been demonstrated in several studies [27,28,29,30]. However, the effect of alternation is more controversial because the studies conducted on this subject, like ours, have no uniform protocol [31,32].

Injuries could be induced by intubation tubes. Nasal intubations are used in our unit, as in all French NICUs. There have only been a few older studies on this subject [33,34,35]. Only one injury occurred with a high-flow nasal cannula in our study, which confirms the results of the literature on the safety of this ventilatory mode [36,37,38,39,40,41,42,43]. Nevertheless, some studies describe a lower rate of nasal injury with this interface versus masks and nasal prongs [44,45,46].

### 4.6. Evolution of Injuries and Determinants

Injury evolution during the first month of follow-up was unfavorable in more than half of the cases. Healing usually occurs within the first 7 days. Therefore, the maximum attention must be paid to injuries as soon as they occur. The secondary protective measures—more specifically, the application of a hydrocolloid—were not effective in improving the outcomes of injuries. When used prophylactically, hydrocolloid was described as a protective factor [47,48]. Used curatively, it seems to lose its benefits.

At discharge, more than half of the injuries had resulted in sequelae, still present at one year. The cases cited were the most serious, requiring surgical treatment [21,49,50,51,52,53,54].

### 4.7. Clinical Implications and Perspectives

Given the frequency of nasal injuries in preterm infants and their short- and medium-term impacts, the skin condition must be monitored when respiratory support is necessary. Primary prevention must be a priority. Industrial innovation and the improvement of CPAP-type interfaces will likely lead to improved skin tolerance. Some systems may have different shapes, thus helping to avoid such possibly frequent and serious injuries.

Studies are underway to develop individual nasal masks by using 3D printers [55] or to test equipment to improve injury detection [56]. While waiting for these results, establishing a protocol for the monitoring of the nasal skin condition could help to raise awareness of this issue among nursing teams [57,58,59,60]. Educating parents and increasing their awareness could help caregivers to identify an altered skin condition as quickly as possible. Moreover, as several studies have shown [61,62,63], a reminder of the correct use of devices, the choice of the interface size and the correct positioning of the interface and its fastening system can reduce the risk of these injuries.

We could speculate that the observed lesions may be an expression of an application problem related to the CPAP device used or to the shape of the nasal prongs. However, this study was not designed to evaluate the mechanisms involved in the development of nasal lesions and whether some types of CPAP devices or interfaces are more likely to trigger these lesions. This deserves further specific studies.

### 4.8. Research Perspectives

A new survey of nasal injuries after the implementation of the French Group of Reflection and Evaluation of the Environment of Newborns (GREEN) recommendations, not implemented in our center at the time of the study, could be interesting [64]. Exhaustive follow-up of such injuries would also be interesting in providing new data on the evolution of the injuries in childhood, because the question of a potential impairment in quality of life may be raised. Long-term follow-up would also reveal how many children undergo surgery and the impact on the parents and the child’s mental health.

## 5. Conclusions

Nasal injuries related to contact with respiratory support interfaces are frequent in preterm infants and, in half of all cases, result in scars that are visible as soon as the patient is discharged from hospital and persist for months afterwards. Parents reported social and esthetic impacts. Once the injury has appeared, it is difficult to heal. Particular attention must be paid to injury prevention, which involves providing information to caregivers and parents, as well as adapted and specific nursing care. The good use of CPAP devices and of nasal interfaces must be a priority. The team must also be well trained in the use of such devices.

## Figures and Tables

**Table 1:** Demographic characteristics of the study population.

Gestational age (weeks^days^), median (range)	27^6/7^ (234/7–32^6/7^)
Birth weight (g), median (range)	986 (485–2090)
Small for gestational age, n/N infants (%)	7/64 (10.9)
Sex, n Male/female	29/35
Delivery mode, n Vaginal/C section	19/45
Apgar, median (range)	
At 1 min	5 (0–10)
At 5 min	8 (1–10)
Antenatal corticosteroid therapy, n/N infants (%)	50/64 (78.1)
Skin phenotype according to Fitzpatrick classification, n/N infants (%)	
I–III	39/64 (60.9)
IV	17/64 (26.6)
V–VI	8/64 (12.5)
Incubator humidification rate (%), median (range)	72 (0–95)
Insufflation air heater temperature (°C), median (range)	37 (35–39)
Total ventilation duration (days), median (range)	
Invasive	2.5 (0–24)
Continuous positive airway pressure	37 (3–68)
Mask	26 (0–53)
Prongs	10 (0–25)
Ram cannula	0 (0–10)
High-flow nasal cannulas	11 (0–31)
Bronchopulmonary dysplasia, n/N infants (%)	25/64 (39)
Duration of caffeine treatment (days), median (range)	58 (13–107)
Duration of doxapram treatment (days), median (range)	0 (0–61)

**Table 2:** Determinants of favorable injury evolution in the first month in preterm infants requiring ventilatory support.

	Favorable Evolution					
	Day 3	*p*	Day 7	*p*	Day 28	*p*
Total, n/N infants (%)	22/64 (34.4)	-	29/63 (46)	-	24/60 (40)	-
Type of ventilation at time of analysis, n/N infants (%)		0.10		0.41		0.04
Invasive ventilation	1/11 (9)		4/9 (44.4)		1/5 (20)	0.58
Continuous positive airway pressure	20/49 (40.8)		20/47 (42.6)		7/27 (25.9)	0.06
High-flow nasal cannulas or ventilator weaning	1/4 (25)		5/7 (71.4)		16/28 (57.1)	0.64
Main injury site, n/N infants (%)		0.12		0.26		0.009
Columella	8/26 (30.8)		10/23 (43.5)		9/27 (33.3)	0.15
Internal part of the nostril orifice	4/19 (21.1)		6/19 (31.6)		5/16 (31.3)	0.22
External part of the nostril orifice	1/2 (50)		1/3 (33.3)		0/1 (0)	1
Intranasal mucosa	3/7 (42.9)		5/7 (71.4)		3/7 (42.9)	1
Cartilaginous dorsum	6/8 (75)		6/8 (75)		7/7 (100)	0.047
Philtrum	0/1 (0)		0/1 (0)		0/0 (0)	1
Other	0/1 (0)		1/2 (50)		0/2 (0)	0.47
Secondary protection measure, n/N infants (%)		0.88		0.68		0.056
None	9/27 (33.3)		16/33 (43.3)		22/47 (46.8)	
Presence	13/37 (35.1)		13/30 (48.5)		2/13 (15.4)	
Hydrocolloid	8/23 (34.8)	0.23	4/15 (26.7)	0.20	1/4 (25)	0.58

**Table 3:** Factors associated with sequelae at discharge.

	Sequelae	No Sequelae	*p*
Main injuries, n/N infants (%)	34/58 (58.6)	24/58 (41.4)	0.28
Total injuries, n/N injuries (%)	48/97 (49.5)	49/97 (50.5)	0.93
Type of ventilation at injury detection, n/N infants (%)			1
Continuous positive airway pressure	29/50 (58)	21/50 (42)	
Invasive ventilation	4/7 (57.1)	3/7 (42.9)	
High-flow nasal cannulas	1/1 (100)	0/1 (0)	
Initial ventilation time (days), mean (SD)			
Continuous positive airway pressure	9.3 (5.7)	10.8 (10.1)	0.71
Invasive ventilation	7.8 (6)	7.2 (2.9)	0.09
Ventilation duration (days)			
Total, median (range)	61 (11–101)	44 (10–85)	0.02
Invasive ventilation, median (range)	2.5 (0–24)	3 (0–24)	0.37
Continuous positive airway pressure, mean (SD)	43.8 (12.9)	29.9 (13)	<0.001
Total non-invasive, mean (SD)	54.7 (14.7)	41.4 (14.2)	0.005
Site, n/N injuries (%)			0.09
Internal part of the nostril orifice	10/22 (45.5)	12/22 (54.5)	
External part of the nostril orifice	2/5 (40)	3/5 (60)	
Columella	21/36 (58.3)	15/36 (41.7)	
Philtrum	0/1 (0)	1/1 (100)	
Cartilaginous dorsum	3/14 (21.4)	11/14 (78.6)	
Intranasal mucosa	10/17 (58.8)	7/17 (41.2)	
Other	2/2 (100)	0/2 (0)	
Severity of injury at inclusion, n/N infants (%)			0.25
Stage 1	11/24 (45.8)	13/24 (54.2)	
Stage 2	19/29 (65.5)	10/29 (34.5)	
Stage 3	4/5 (80)	1/5 (20)	
Maximum severity of injury, n/N infants (%)			<0.001
Stage 1	2/11 (18.2)	9/11 (81.8)	0.13
Stage 2	5/19 (26.3)	14/19 (73.7)	0.09
Stage 3	27/28 (96.4)	1/28 (3.6)	<0.001
Secondary protection measure, n/N infants (%)			0.08
Presence	27/41 (65.9)	14/41 (34.1)	
None	7/17 (41.2)	10/17 (58.8)	
Hydrocolloid, n/N infants (%)			0.03
Presence	18/24 (75)	6/24 (25)	0.04
None	16/34 (47.1)	18/34 (52.9)	0.78
Birth weight (g), median (range)	957 (560–1544)	1047 (485–2090)	0.06
Growth, n/N infants (%)			1
Small for gestational age	4/6 (66.7)	2/6 (33.3)	
Eutrophic	30/52 (57.7)	22/52 (42.3)	
Age at inclusion (days), median (range)	10.5 (1–38)	11 (1–49)	0.45
Gestational age (weeks^days^), median (range)	27^4/7^ (23^4/7^–32^2/7^)	28^4/7^ (23^4/7^–32^6/7^)	0.06
Skin phenotype, n/N infants (%)			0.93
I–III	19/34 (55.9)	15/34 (44.1)	
IV	10/16 (62.5)	6/16 (37.5)	
V–VI	5/8 (62.5)	3/8 (37.5)	
Sex, n/N infants (%)			0.37
Female	21/33 (63.6)	12/33 (36.4)	
Male	13/25 (52)	12/25 (48)	

## Data Availability

The original contributions presented in this study are included in the article/Supplementary Materials. Further inquiries can be directed to the corresponding author.

## Supplementary Materials

The following supporting information can be downloaded at: https://www.mdpi.com/article/10.3390/children12070840/s1, Table S1. Characteristics of injuries at inclusion according to ventilatory support interface responsible. Table S2. Characteristic of injuries at inclusion according to continuous positive airway pressure interface. Document S1: Nasal injury assessment and monitoring tool. Document S2: Parental questionnaire.
