# Supplementary material for: Nasal Injuries Related to Respiratory Support Interfaces in Preterm Infants: Neonatal Course and 12-Month Outcome
Source: Children (Basel). 2025 Jun 26;12(7):840. doi: 10.3390/children12070840 (PMC12293294; doi:10.3390/children12070840)
Supplement: Supplementary file 1 [file children-12-00840-s001.zip › children-3697803-supplementary.pdf]

Table S1. Characteristics of injuries at inclusion according to ventilatory support interface responsible.

	Invasive ventilation	Continuous positive airway pressure	p
**Main injuries, n/N infants (%)**	10/64 (15,6)	53/64 (82,8)	< 0.001
**Total injuries, n/N injuries (%)**	11/79 (13,9)	67/79 (84,8)	< 0.001
**Severity of the main injury, n/N infants (%)**			
Stage 1	4/10 (40)	20/53 (37,7)	NS
Stage 2	5/10 (50)	29/53 (54,7)	
Stage 3	1/10 (10)	4/53 (7,5)	
**Site, n/N injury (%)**			< 0.01
Columella	1/11 (9,1)	25/67 (37,3)	NS
Internal part of the nostril orifice	9/11 (81,8)	12/67 (17,9)	< 0.01
External part of the nostril orifice	1/11 (9,1)	4/67 (6)	NS
Philtrum	0/11 (0)	1/67 (1,5)	NS
Cartilaginous dorsum	0/11 (0)	10/67 (14,9)	NS
Intranasal mucosa	0/11 (0)	14/67 (20,9)	NS
Other	0/11 (0)	1/67 (1,5)	NS

Table S2. Characteristic of injuries at inclusion according to continuous positive airway pressure interface.

	Nasal Mask	Nasal prongs (alternation mask)	p
**Infants, n/N infants (%)**	36/53 (67,9)	17/53 (32,1)	0.03
**Injuries, n/N injuries (%)**	43/67 (64,2)	24/67 (35,8)	0.06
**Alternation of interface, n/N infants (%)**			
None	3/36 (8,3)	0/17 (0)	
Interface size	5/36 (13,9)	0/17 (0)	NS
Interface type	6/36 (16,7)	5/17 (29,4)	
Interface type and size	22/36 (61,1)	12/17 (70,6)	
**Severity of the main injury, n/N infants (%)**			
Stage 1	15/36 (41,7)	5/17 (29,4)	NS
Stage 2	18/36 (50)	11/17 (64,7)	
Stage 3	3/36 (8,3)	1/17 (5,9)	
**Site, n/N injury (%)**			
Columella	16/43 (37,2)	9/24 (37,5)	
Internal part of the nostril orifice	7/43 (16,3)	5/24 (20,8)	
External part of the nostril orifice	3/43 (7)	1/24 (4,2)	
Philtrum	1/43 (2,3)	0/24 (0)	NS
Cartilaginous dorsum	9/43 (20,9)	1/24 (4,2)	
Intranasal mucosa	6/43 (14)	8/24 (33,3)	
Other	1/43 (2,3)	0/24 (0)	

Document S1: Nasal injury assessment and monitoring tool

Short to medium-term evolution of nasal skin lesions related to ventilation interfaces discovered during the neonatal period in very preterm infants

Day O: discovery of the lesion

-Date :
-Name et first name of newborn:
-Date of birth:
- Gestational age (weeks, days) :
- Current type of ventilation interface and maximum fio2 :
- Number of days of ventolatory support:
- Pommade / protective interface : yes / no (if yes, what type)

1/ TYPE OF LESION (according to Fischer stage)
Erythèma (A) ? Erosion, bleeding, superficial ulceration (B) ? Loss of substance (C) ? Nécrosis (C)

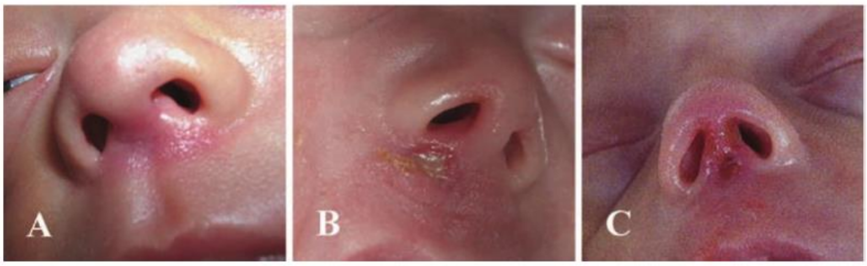

(Photos taken from Fischer et al., 2010a)

2 /LOCALISATION OF THE LESION (mark the area with a cross) :

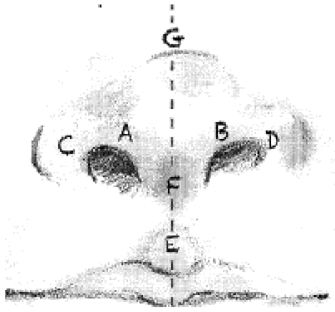

FOLLOW UP: At DAY 3, 7, 28, Discharge, Immunisations : 2nd month/ 3rd month

Location	Type of lesion according to Fischer stage (a-b-c)	Ventilatory support + Fio2 Treatment modification	Loss of substance: Yes - no	Narinary dilatation / enlargement Yes - no
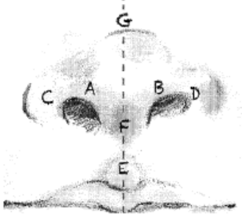				

FOLLOW UP: At 4 months of corrected age, 9 months of corrected age and 12 months of corrected age

- Hospital : hautepierre / mulhouse / saverne / colmar/ haguenau
- Date :
- Name and first name :

LOCATION OF THE LESION
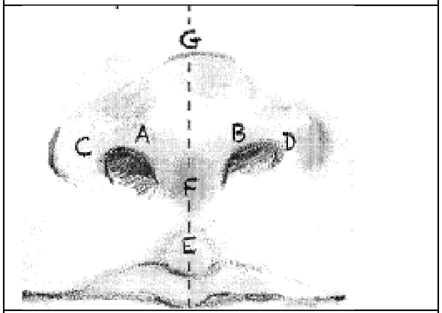

AESTHETIC ANOMALY
- Change in the appearance of the nose: YES-NO<br>- loss of substance: YES-NO<br>- dilation / enlargement of the nose: YES-NO<br>- Change in pigmentation: YES-NO<br>- Change in skin thickness: YES-NO
FUNCTIONAL ABNORMALITY<br>- Respiratory sounds: YES-NO<br>- Deviation of the nasal septum: YES-NO

Document S2: Parental questionnaire

Parental Questionnaire

This questionnaire aims to gather each parent's opinion regarding their perception of the nasal lesion being monitored in their child as part of the ongoing study: "The medium-term evolution of nasal skin lesions related to ventilation interfaces discovered during the neonatal period."

1) In your opinion, does your child have a deformation of the nasal region?
a) Agree
b) Neutral
c) Disagree

2) In your opinion, does your child have a loss of skin substance?
a) Agree
b) Neutral
c) Disagree

3) In your opinion, does your child have dilation/enlargement of the nostrils?
a) Agree
b) Neutral
c) Disagree

4) In your opinion, does your child have a change in pigmentation?
a) Agree
b) Neutral
c) Disagree

5) In your opinion, does your child have a change in skin thickness?
a) Agree
b) Neutral
c) Disagree

6) In your opinion, does your child make respiratory noises related to this condition?
a) Agree
b) Neutral
c) Disagree

7) Have you received any comments from people around you regarding this condition?
a) Agree
b) Neutral
c) Disagree

8) Have you considered the possibility of aesthetic treatment in the future?
a) Agree
b) Neutral
c) Disagree
